# Economic modelling of providing ‘spare’ adrenaline autoinjectors to all schools to improve the management of anaphylaxis

**DOI:** 10.1136/archdischild-2025-329493

**Published:** 2025-10-21

**Authors:** Paul J. Turner, Andrew Bright, Louise J. Michaelis, Jennifer K. Quint

**Affiliations:** 1 National Heart & Lung Institute, Imperial College London, London, UK; 2 Gateshead Health NHS Foundation Trust, Gateshead, UK; 3 Paediatric Immunology and Allergy, Great North Children’s Hospital, Newcastle Upon Tyne, UK; 4 Population Health Sciences Institute, Newcastle University, Newcastle upon Tyne, UK; 5 School of Public Health, Imperial College London, London, UK

**Keywords:** Allergy and Immunology, Child Welfare, Emergency Care, Epidemiology, Health Care Economics and Organizations

## Abstract

**Objective:**

To analyse NHS health datasets to estimate the cost of providing emergency adrenaline [epinephrine] autoinjectors (AAIs) to school pupils on a named-patient basis to leave on school premises versus providing ‘spare’ AAIs to schools which can be used for any school pupil.

**Design:**

Retrospective cohort study.

**Setting:**

English primary electronic health data from the Clinical Practice Research Datalink (CPRD) and English prescriptions data from the NHS Business Services Authority.

**Participants:**

School-aged children in England.

**Main outcome measures:**

(1) Proportion of school children with food allergy prescribed AAI; (2) cost of providing more than two AAIs to individual pupils mapped to integrated care boards (ICBs) in England compared with the cost of providing four spare AAIs to every school for the academic year 2023/24.

**Results:**

44% of school-aged children in the CPRD had at least one AAI prescription and only 34% had repeat AAIs prescribed. In pupils with previous anaphylaxis, rates were 59% and 44%, respectively. During the academic year 2023/24, 63% of pupils were dispensed more than two AAIs at an estimated cost of over £9 million. The estimated cost of providing spare AAIs to every school was £4.5 million. If spare AAIs were to replace the supply of named-patient AAIs exclusively to leave on school premises, this would represent a potential cost-saving of at least £4.6 million or 25% of the total national expenditure for AAIs.

**Conclusions:**

Under half of children at risk of anaphylaxis are prescribed AAIs. Providing spare AAIs to all schools (at no cost to the school) would be a cost-neutral strategy for the vast majority of ICBs and one that is likely to improve emergency access to AAIs and therefore safety.

**WHAT IS ALREADY KNOWN ON THIS TOPIC**

- In 2017, UK legislation was changed to allow schools to obtain, without prescription, ‘spare’ adrenaline [epinephrine] autoinjector (AAI) devices for the emergency treatment of anaphylaxis in any school pupil.

**WHAT THIS STUDY ADDS**

- Under half of school-aged children with food allergy (and at potential risk of anaphylaxis) are prescribed AAIs.
- Although the Medicines and Healthcare Products Regulatory Agency recommends people at risk of anaphylaxis carry two AAIs, over 60% of school-children prescribed AAIs were dispensed more than four AAIs in the academic year 2023/24; it is likely that the majority of these additional AAIs were provided to be left on school premises.
- If spare AAIs were provided to all schools, to avoid the need for pupils to leave their own AAIs on school premises, this would represent a potential cost-saving of at least £4.6 million or 25% of the total national expenditure for AAIs.

**HOW THIS STUDY MIGHT AFFECT RESEARCH, PRACTICE OR POLICY**

- This analysis clearly demonstrates that providing spare AAIs to schools (at no cost to the school) would be a cost-neutral strategy that would improve emergency access to AAIs for all school pupils (not just the minority prescribed AAIs) and also increase the resilience of the UK supply chain for AAIs.

## Introduction

 Around 3% of school-aged children in the UK have immunoglobulin E (IgE)-mediated food allergies.^1^ Thus, on average, UK school classes will have one or two children at risk of food-induced anaphylaxis, a serious allergic reaction which may be life-threatening. Even with the best dietary avoidance, most children will have at least one accidental reaction every 2–3 years.^2^,^4^ While most will not progress to anaphylaxis, severity is unpredictable, which is why people at risk of anaphylaxis are usually prescribed adrenaline [epinephrine] autoinjectors (AAIs) for emergency use.^5^ The majority of reactions respond to a single dose, but up to 10% require a further dose^6^ and devices may misfire or be used incorrectly. This is why the UK Medicines and Healthcare Products Regulatory Agency (MHRA) and European Medicines Authority recommend that individuals at risk of food anaphylaxis have access to two AAIs at all times.^7 8^

School children spend around 20% of their waking hours in school. It is therefore not surprising that 16–18% of school-aged children with food allergies have had a reaction in school.^9 10^ Around 80% of all anaphylaxis reactions to food occur in school-aged children,^11^ and 10% of these happen at school.^12 13^ One quarter of these anaphylaxis reactions in school occur in pupils with no prior allergy diagnosis.^14^ Fortunately, fatal anaphylaxis is rare,^15^ but it is also very unpredictable^5^: 17% of anaphylaxis deaths in school-aged children in the UK happen in the educational setting.^15^

To help mitigate this risk, many schools require pupils at risk of anaphylaxis to not only have AAIs with them, but to leave the devices on school premises in case they forget to bring them in. While the MHRA is explicit about the need to carry two AAIs at all times,^7^ there is less clarity over the number of devices that should be prescribed to school children: the British Society for Allergy & Clinical Immunology (BSACI) is increasingly aware of general practitioners who refuse to prescribe more than two devices to any given individual. Many children with food allergies who have had only mild reactions previously are not prescribed AAIs; however, anaphylaxis often happens in those with only previous mild reactions.^5^ Under current UK legislation, an AAI supplied on prescription to any given patient cannot be used in someone else—even in an emergency^16^—so schools can only use a child’s AAI in that specific child.

In 2015, the BSACI, working with the patient charities Anaphylaxis UK and Allergy UK, undertook a national survey to evaluate anaphylaxis care in schools. Responses were received from 1609 parents and 821 teachers, with representation from every region across the UK.^17^ Parents reported that 83% of children with food allergies had been prescribed AAIs to leave on school premises (the majority had two devices, although 18% were issued with a single device and 10% were supplied with three or more devices specifically for school). A total of 93% of teachers worked in a school with at least one child prescribed AAIs for school.

It is this background that led to UK law being changed in 2017 to allow for schools to obtain, without a prescription, ‘spare’ AAI devices for use in emergencies (for example, when the pupil’s own AAI is not readily available or they do not have their own AAI prescribed).^18^ To support schools, the Department of Health and Social Care (DHSC), together with key stakeholders, developed non-statutory guidance.^19^ The guidance recommended that pupils in secondary school (year 7) should keep their own prescribed AAIs with them at all times. However, uptake of spare AAIs has been limited, with only around half of schools doing so.^20^ This may be because schools have to fund the cost of spare AAIs directly and pay ‘market rate’ – often in excess of £100 per device (rather than the subsidised NHS tariff, currently £9.90 for two devices).

To address this, some integrated care boards (ICBs) have funded local pilots whereby spare AAIs are provided to local schools.^21^ We analysed NHS health datasets to assess the potential cost of providing spare AAIs to schools, and how this might be offset by primary care no longer providing AAIs to individual pupils (on a named-patient basis) to leave on school premises.

## Methods

### Data sources and study population

The Clinical Practice Research Datalink (CPRD) Aurum is a large UK primary care electronic healthcare record database with current data for approximately 20% of the English population; it is considered representative of the English population in terms of age, sex, deprivation and regional distribution.^22^ We used data from the May 2021 build of the CPRD Aurum (including AAI prescription data) and secondary care data from NHS England’s Hospital Episode Statistics (HES) Admitted Patient Care database to evaluate prescription data for AAIs in children ≤18 years between 2008 and 2018.^1^

The NHS Business Services Authority (NHSBSA) is an arm’s length body of the DHSC, delivering a range of national platforms, systems and services to support primary care, the NHS workforce and UK citizens. NHSBSA processes around 1.1 billion NHS prescription items annually, dispensed within a primary care setting. We evaluated NHS prescriptions data relating to AAIs from April 2022 to March 2025. The data were limited to prescribing in primary care in England, which is also dispensed in the community in England. Further information regarding the dataset and caveats over its use can be found in the online supplemental methods.

### Analyses

We evaluated children and young people aged 5–18 years with a diagnosis of food allergy in the CPRD Aurum, as previously described.^1^ Linking this to 2015 Index of Multiple Deprivation (IMD) data for England and secondary healthcare data from the HES Admitted Patient Care database, we evaluated potential factors associated with AAI prescriptions using a logistic regression model to estimate odds ratios (GraphPad Prism, version 10.4.2).

We then evaluated AAI prescriptions issued to school pupils of primary (reception–year 6) and secondary school age (year 7–year 11) during the 2023/24 and 2024/25 academic years using NHSBSA data. Specifically, we assessed the number of pupils prescribed more than two AAIs in the period of interest in England as a whole and by ICB. Children who weigh around 25 kg are often switched from a 150 μg to a 300 μg dose: we therefore excluded devices prescribed prior to a change in prescription dose when this was observed within the year of interest. For the most recent academic year 2024/25, data were only available for the 8 months from August 2024 to March 2025; therefore, we estimated the annual cost by extrapolating the data to a 12-month period. We assessed the validity of this approach by evaluating monthly dispensing of AAIs, and also applying this method to the academic year 2023/24 where data were available for the full 12-month period.

We estimated the potential annual cost-savings, both overall and by ICB, if ICBs were to provide every school in England with four spare AAIs on an annual basis (for primary schools, two 150 μg doses and two 300 μg doses in line with DHSC guidance;^19^ for secondary schools, four 300 μg doses) rather than supply more than two AAIs to each pupil prescribed AAIs in a given year. NHSBSA prescription data do not show why a patient was prescribed an AAI—it could be that they are replacing expired, misplaced or used devices, or provided as additional sets for other settings rather than being supplied as additional AAIs for school use. Given that the in-date period for AAIs is usually at least 12 months, we used a base assumption that dispensing more than two AAIs was for an additional supply for school (because replacement AAIs for used devices are typically dispensed through hospital pharmacies).^23^ However, we also ran a sensitivity analysis where we assumed that dispensing a single device was more likely to be a replacement while dispensing a pair of devices (two AAIs) was more likely to be for school because schools typically request two AAIs to be left on the premises per pupil.^17^ This assumption is supported by data showing that 90% of reactions respond to a single dose of epinephrine.^6^ Using this approach, we therefore calculated an estimated minimum and maximum cost-saving. Data relating to the number of schools within each ICB were obtained from the Office for National Statistics (ONS).^24^ We assumed the same cost for supplying AAIs to schools as to patients, given that currently, supply to schools of spare pens is through community pharmacies.^19^

Finally, we explored potential factors to explain why the cost/savings of supplying spare pens might vary from one ICB to another. We evaluated the following factors using a logistic regression model: degree of urbanisation (proportion of lower layer super output areas (LSOAs) within an ICB categorised as urban by the ONS);^24^ mean IMD score for ICB; recorded ethnicity as white British or non-white within each ICB (data from ONS); proportion of school pupils dispensed any, at least three or at least four AAIs; number of schools per 100 000 children within the ICB.^24^

## Results

### Frequency of any AAI prescription in school-aged children in CPRD Aurum

A total of 28 520 children and young people aged 5–18 years (inclusive) had at least one diagnostic code for food allergy in the CPRD Aurum and were eligible for linkage with HES data; 21 586 met the definition for probable food allergy.^1^ Overall, 9567 (44%) had at least one AAI prescribed (online supplemental Table S1). AAI prescriptions were more common in children of primary school age (49% of 5–10 year olds) compared with those of secondary school age (40% of 11–18 year olds, p<0.0001, χ^2^). Only 34% (40% of 5–10 years, 28% of 11–18 years; p<0.0001) had a repeat prescription for AAIs. Nut allergy and a history of previous anaphylaxis were associated with a higher odds of AAI prescription, while increasing age and higher IMD were associated with lower odds (table 1). Being managed exclusively outside the hospital setting was associated with a slightly lower OR for AAI prescription (OR 0.87, p=0.01), but not for repeat prescription.

**Table 1:** Factors associated with AAI prescriptions in the Clinical Practice Research Datalink (CPRD) dataset

Factor	At least one prescription for AAI	Repeat prescription for AAI
Age	0.95 (0.94 to 0.95)*	0.92 (0.91 to 0.93)*
Sex	1.02 (0.96 to 1.08)	1.00 (0.95 to 1.07)
IMD	0.95 (0.93 to 0.96)*	0.93 (0.91 to 0.95)*
Nut allergy	6.14 (5.64 to 6.69)*	6.04 (5.48 to 6.67)*
History of previous anaphylaxis	4.09 (3.50 to 4.81)*	3.47 (2.96 to 4.06)*
Managed exclusively in primary care	0.87 (0.78 to 0.97)	0.96 (0.85 to 1.08)

Data are ORs (95% CIs).

* p<0.0001.

IMD, Index of Multiple Deprivation. AAI, adrenaline autoinjector .

### Frequency of prescription of more than two AAIs

Using NHSBSA prescription data, we found that during the 2023/24 academic year, 63% of school-aged children prescribed AAIs were dispensed with at least three devices, and 60% received at least four devices. The proportion of AAI prescription items across the whole population that could not be linked to individual patients was 1.3% between August 2023 and March 2025. The estimated cost of providing more than two AAIs per person was more than £9 million, representing almost half of the total ICB expenditure for AAIs in that year (table 2). Prescription of more than two AAIs was more common for primary school-aged children versus those in secondary school (p<0.0001, χ^2^). Similar patterns were seen for the 8-month period from August 2024 to March 2025. We did not find any significant impact of IMD on rates of dispensing more than two AAIs (data not shown).

**Table 2:** Economic cost of providing spare epinephrine autoinjectors to all schools in England if this cost was offset by the cost of no longer routinely dispensing more than two devices to school-aged children

School type	Individuals with AAIs (n)	Total AAIs dispensed (n)	Estimated total cost of AAIs+applicable fees (£ million)	Individuals with ≥3 AAIs		Individuals with ≥4 AAIs		Estimated annual cost of supplying spare AAIs to schools (£ million)				
									Minimum estimated potential annual savings		Maximum estimated potential annual savings	
				%	Estimated cost of supplying >2 AAIs (£ million)	%	Estimated cost of supplying >2 AAIs (£ million)		£ million	% total budget on AAIs	£ million	% total budget on AAIs
Academic year 2023/24 (children in reception to year 11)*												
All	81 930	327 020	18.3	63	9.2	60	9.0	4.5	4.6	25	4.7	26
Primary	47 330	197 020	11.0	68	5.8	64	5.7	3.8	1.9	17	2.0	18
Secondary	34 590	130 000	7.3	57	3.4	53	3.3	0.7	2.6	36	2.7	37
**Academic year 2024/25 (children in reception to year 11)†**												
All	67 410§	237 810§	14.8§	56§	6.5§	53	6.3§	5.0§				
	101 115‡	356 715‡	22.2‡		9.7‡		9.5‡		4.5‡	20‡	4.8‡	21‡
Primary	39 290§	143 610§	9.0§	60§	4.1§	57	4.0§	4.2§				
	58 935‡	215 415‡	13.4‡		6.1‡		6.0‡		1.8‡	14‡	2.0‡	15‡
Secondary	28 120§	94 200§	5.9§	51§	2.4§	47	2.3§	0.8§				
	42 180‡	141 300‡	8.8‡		3.6‡		3.5‡		2.7‡	31‡	2.8‡	32‡

* Omits 2.7% of items and their associated drug cost where a change in prescribing regime has been observed.

† Omits 3.4% of items and their associated drug cost where a change in prescribing regime has been observed.

‡ Estimated, as raw data only available from August 2024 to March 2025.

§ Data reported for 8 months.

AAI, adrenaline autoinjector.

### Monthly trends in AAI prescribing

**Figure 1:**
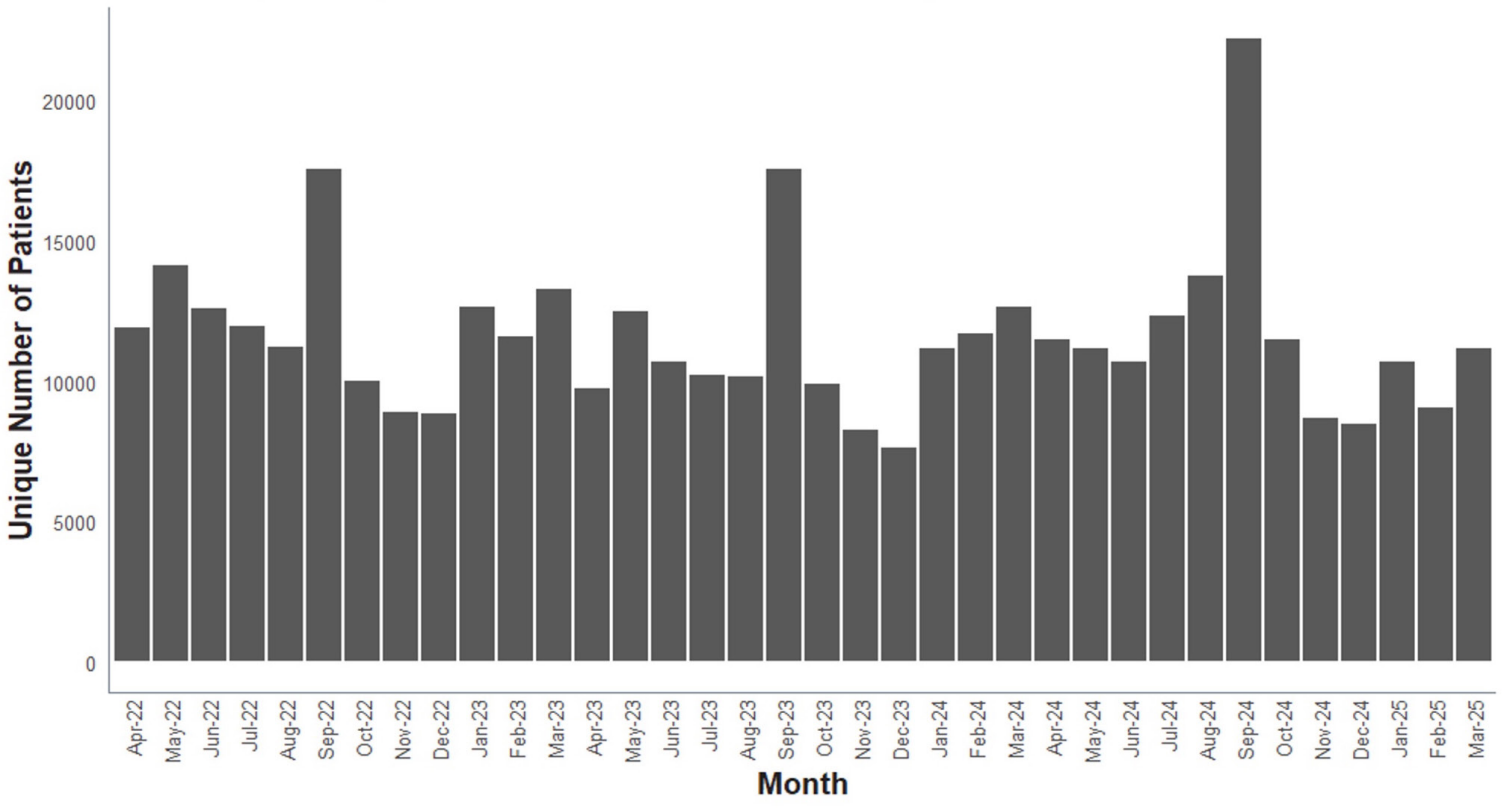
Number of individuals with a prescription for adrenaline autoinjectors dispensed by calendar month.

At the time of analysis, data for the 2024/25 academic year were available for the first 8 months only as the academic year was still in progress. To provide an annual estimate for the cost of providing spare AAIs to schools for 2024/25, we assessed whether it was reasonable to extrapolate data for August 2024–March 2025 to the entire academic year. To test the validity of this approach, we examined if the number of AAIs dispensed each month was consistent across the year from April 2022 to March 2025. We found evidence for a monthly spike in AAI prescriptions dispensed in September (figure 1), coinciding with the start of the UK academic year; 13% of all AAI prescriptions were issued in September, instead of an expected monthly average of 8.3%. Given this, we also evaluated the impact of extrapolating 8 months of data (August 2023–March 2024) to the entire academic year (August 2023–July 2024), and compared this to the actual data available for the 12 months from August 2023 to July 2024. This analysis is shown in online supplemental Table S2. Despite the ‘September spike’, extrapolating data from August 2023 to March 2024 to the full 12-month period provided a reasonable estimate of the number of AAIs prescribed and thus the total cost, but underestimated the proportion of pupils dispensed more than two AAIs.

### Economic modelling of providing spare AAIs

**Figure 2:**
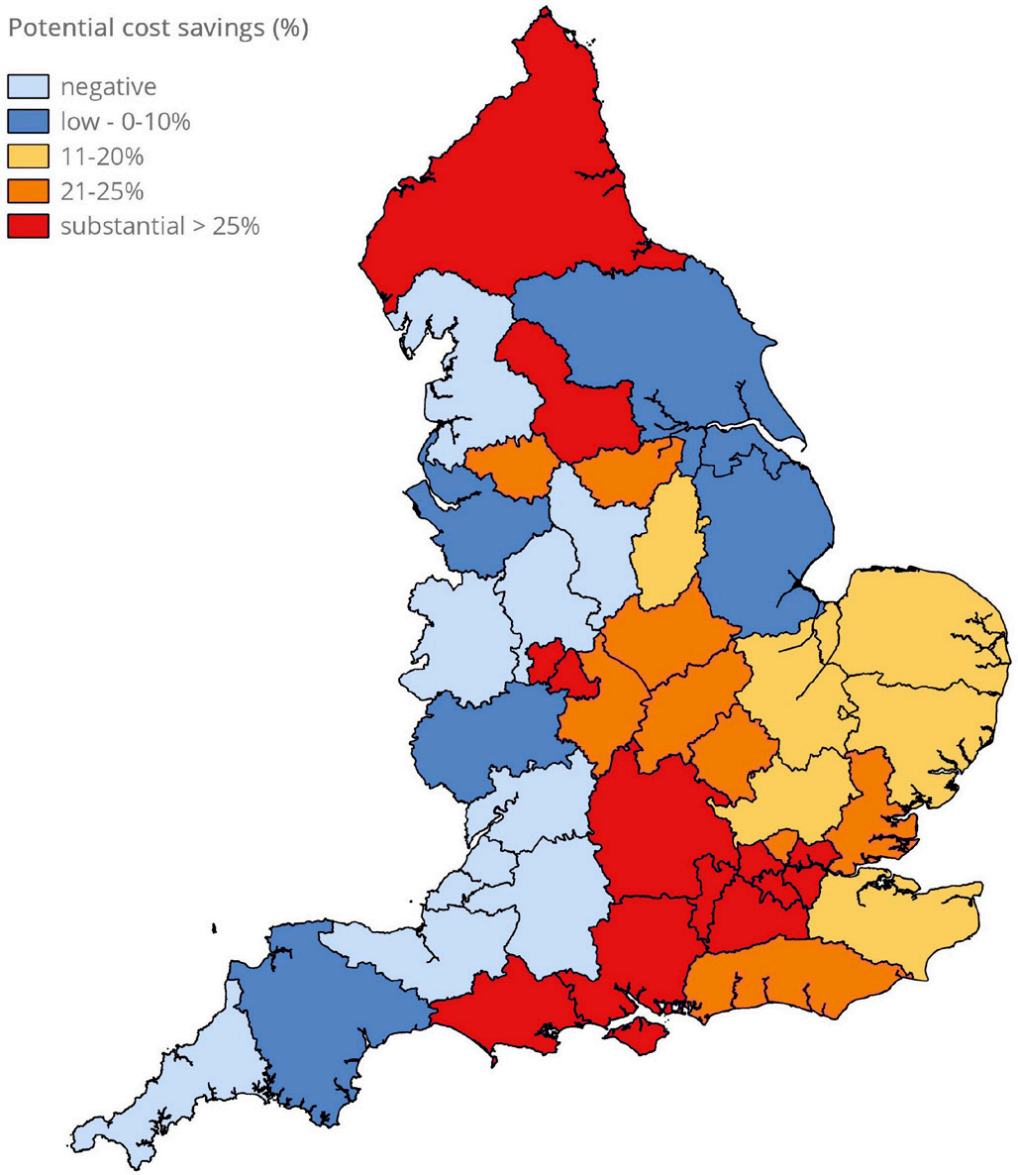
Estimated cost-savings by integrated care board (ICB) by limiting general supply of adrenaline autoinjectors on a named patient basis to two devices per person, and supplying all schools within the ICB with four spare devices for emergency use. Data for the academic year 2023/24, as shown in online supplemental Table S2

Finally, we estimated the relative cost/saving of providing every school in England with four spare AAIs on an annual basis, and whether this could be offset by the current cost of dispensing additional AAIs to pupils beyond the two recommended by the MHRA. For the 2023/24 academic year, the cost of providing spare AAIs to every school was estimated to be £4.5 million; the cost of providing more than two AAIs on a named-patient basis was more than £9 million, therefore this represented a potential cost-saving of at least £4.6 million or 25% of the total national expenditure for AAIs (table 2). Estimated savings by ICB are shown in online supplemental Table S3. Across the 42 ICBs, only four (10%) would incur additional significant cost (≥£10 000, approximately 5% of total expenditure on AAIs) while 31 (74%) would achieve cost-savings in excess of £10 000 (and some in excess of £400 000). The average cost-saving per ICB would be over £70 000 (online supplemental Table S1 and figure 2). A similar level of savings was also noted for the academic year 2024/25 (table 2), with estimated savings by individual ICB shown in online supplemental Table S4.

**Figure 3:**
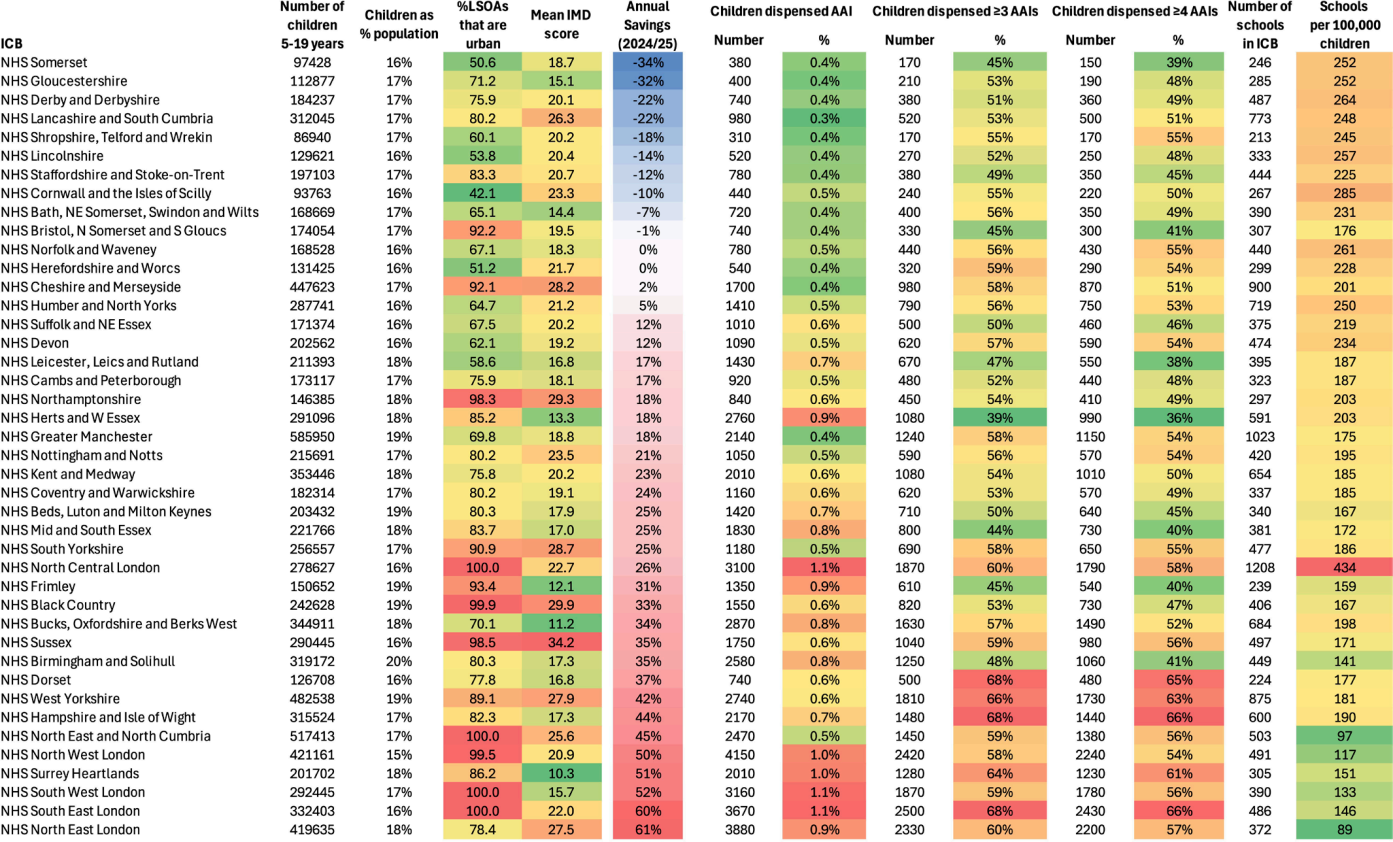
Heat map of estimated cost-savings by integrated care board (ICB) for the academic year 2024/25, by urbanisation, mean index of multiple deprivation (IMD), adrenaline autoinjectors (AAIs) dispensed and schools’ density. Urbanisation is represented by the number of lower layer super output areas (LSOAs) within the ICB that are urban. Demographic data including IMD are for 2021/22. Data sourced from the Office for National Statistics.

Significant differences between the saving/cost of implementing funded spare AAIs were noted between ICBs. We used a heat map (figure 3) to explore the following potential factors: degree of urbanisation; mean IMD score for the ICB; recorded ethnicity; proportion of school pupils dispensed AAIs (including in excess of two devices) and the density of schools within each ICB. With the exception of mean IMD, all factors were significantly associated with the saving/cost (see online supplemental Table S5). To address for confounding, we performed a multivariate analysis using logistic regression (by ordinary least squares): only the proportion of children prescribed (any) AAIs and school density were significant (both p<0.0001).

## Discussion

In this analysis of national datasets in England, almost two thirds of school-aged pupils who are prescribed AAIs are dispensed with more than two devices per annum, with a higher rate in primary school children. While we could not determine the reason for these additional devices, most pupils were dispensed at least another two devices (rather than just a single device). Given that 90% of anaphylaxis reactions respond to a single AAI dose^6^ and that the rate of accidental reactions in nut-allergic children is around 10–15% per annum,^2^,^4^ this suggests that the majority of additional AAIs dispensed were likely to be additional devices for leaving in school, rather than to replace used AAIs. Over half of students in secondary school were also prescribed more than two devices, despite government guidance advising that these students should carry their own prescribed AAIs with them^19^ (and therefore not need additional AAIs just for school use).

Since 2017, UK schools have been able to purchase, without a prescription, spare AAI devices for emergency use to treat anaphylaxis.^18^ These spare devices could be used in any pupil irrespective of whether they had been prescribed AAIs, so long as they had an individualised healthcare plan (IHCP) and parental consent. They are not intended to replace a pupil’s own prescribed AAI, but to provide a ‘back-up’ if these are not readily available in an emergency.^19 25^ Subsequently, the MHRA clarified that spare AAIs could be used in *any* individual (including adults and visitors) in an emergency, but this should be ‘for exceptional circumstances only that could not have been foreseen’.^25^ However, uptake of spare AAIs has been limited,^20^ with the need for schools to pay for the spare AAI themselves as a major factor.^20^ This is in contrast to other schemes in Australia,^26^ Canada^27^ and the USA^28^ where spare AAIs have been funded centrally.

The need for further change has been flagged by two of His Majesty’s Coroners following inquests into the deaths of Mohammad Ismaeel Ashraf^29^ and Karanbir Cheema^30^ in school, as a result of anaphylaxis. Key concerns highlighted included inadequate staff training resulting in delayed and incorrect administration of epinephrine, and a failure to ensure AAIs were in-date and accessible in an emergency—issues which can be addressed through mandatory provision of spare AAIs and training.^31^

For most ICBs, we estimated that the cost of spare AAIs to all schools could be fully offset by the ICB no longer funding AAIs on a named-patient basis exclusively for school use—something entirely consistent with both current legislation^18^ and guidance from the MHRA.^32^ There are additional reasons to support such a strategy. Less than 10% of children with food allergies are seen in a specialist allergy clinic^1^; therefore, many children may not have their risk of anaphylaxis assessed by someone with the requisite experience. In a 2012 survey of 2439 school nurses in the USA, 25.3% of students with food allergies had no AAI and only 24.6% had two unexpired devices at school.^33^ In a survey of 5683 US schools in 2013/14, 607 (11%) reported 919 anaphylaxis events; 22% happened in pupils with no known allergies. Fifty-four pupils (9%) received a second AAI dose.^14^ The NSW Anaphylaxis Education Program in Australia was established in 2004 to improve state-wide anaphylaxis care following several deaths due to anaphylaxis in schools. This included providing spare AAIs to all schools.^34^ Between 2017 and 2019, 341 students had anaphylaxis, of whom 130 (38%) were treated with a spare AAI.^35^ Reasons for using the spare AAI included: AAI prescribed but not with the child in school or expired (n=17, 5% of anaphylaxis events); no known prior allergies (77, 23%); or known diagnosis of food allergy but AAI not prescribed (36, 11%). By providing all schools with spare AAIs, all school pupils will be able to access potentially life-saving adrenaline in an emergency.

A further benefit, noted by the DHSC, is that providing spare AAIs to schools allows schools to hold just a single brand of AAI and avoids the school having to have multiple devices produced by different manufacturers; this reduces confusion over how to use the device (given that instructions differ between brands).^19^ In an emergency, staff can waste valuable minutes identifying a child’s own AAIs, since they cannot use those belonging to someone else. The presence of different brands of AAIs can be confusing, leading to delays in administration as flagged in some inquests.^29 30^ Providing spare AAIs reduces the time wasted in trying to identify a given child’s own AAI in an emergency situation where minutes can matter and delays in treatment are associated with fatal outcomes.^31^

Not every ICB would achieve a relative cost-saving with this strategy. The majority of ICBs where additional cost would be incurred (rather than a saving) were in West England. Exploring potential reasons for this, the two most important factors were the number of children prescribed AAIs (which reflects the number of children with food allergy) and the number of schools within the ICB. While these areas also tend to have lower proportions of people from non-white backgrounds, the impact of this is likely to be due to rates of food allergy rather than ethnicity, since a multivariate analysis showed that ethnicity was not an independent factor. The link between ethnicity and increased risk of food-related anaphylaxis has been documented.^36^ While we found that IMD is associated with lower rates of AAI prescription in children with food allergy, ‘mean’ IMD lacks the granularity needed for this to impact on AAIs dispensed at a summary IMD level. Likewise, while savings were lower in more rural areas, this is reflected in the density of schools within the ICB.

Our analysis is not without limitations: there are a number of caveats regarding the use of the NHSBSA data. Primarily, the dataset only includes primary care NHS prescriptions in England and dispensed by community pharmacies and excludes AAIs dispensed through hospitals and private healthcare. We were not able to extend this analysis to Scotland, Wales and Northern Ireland. However, we note the existing evidence for very limited uptake of spare AAIs in Wales and suspect the findings of our analysis are valid throughout the UK.^37^ We could not analyse the reason for dispensing more than two AAIs to any given individual; therefore, we cannot determine whether additional AAIs were dispensed to replace expired or lost devices, or to be used in other settings, or to supply additional devices for school use. Notwithstanding, given the spike in the number of AAIs dispensed at the beginning of the school year, and that most of these are for two or more devices (rather than single devices), it is likely that the majority of additional AAIs were for school use rather than to replace used devices.

Schools currently obtain spare AAIs by placing a request through local pharmacies.^19^ We could therefore assume that the cost of providing spare AAIs to schools was equivalent to those dispensed on a per-patient basis. If ICBs were to provide spare AAIs, this might occur through an alternative distribution arrangement, which could affect cost. However, there may be significant advantages: centralised supply may allow schools to be issued AAIs from the same batch, meaning the devices would have the same expiry date. This would reduce the burden on schools and allow for more systematic replacement. Centralised distribution would also facilitate monitoring of allergic reactions in school and help learning from incidents (something already required by UK legislation): such a system has been critical to the success of the NSW Anaphylaxis Education Programme in Australia, improving the care of students with allergy in schools.^34 35^ Mandatory education of school staff is an essential part of the scheme—an ongoing issue in the UK, which has also been repeatedly flagged as a concern.^20 21 29 30^

Irrespective, there can be little doubt that if ICBs were to limit dispensing to two unexpired AAIs per pupil at any one time (and so no longer provide additional AAIs on a named-patient basis just for school use), then providing spare AAIs to schools (at no cost to the school) would be a cost-neutral strategy for the vast majority of ICBs—and one that is likely to improve emergency access to AAIs and therefore safety. This would also increase the resilience of the UK supply chain for AAIs (something which has been a major concern in the past decade, and a contributory factor in at least one fatality)^38^ and reduce wastage. While over 2.3 million AAI devices are sold each year in the UK, only around 2% are actually used.^7^ In this respect, the recent approval of an intranasal adrenaline device might be advantageous since the shelf-life is longer than for AAIs^39^ (as well as avoiding the issues over needle phobia), although whether intranasal adrenaline is as effective as AAIs is currently unclear.^40^

In 2020, an editorial concluded that providing spare AAIs to schools can ‘be achieved with minimal cost implications: with mandatory “spare” AAI provision, families would no longer need to provide the school with a supply of AAIs for each child, something which would avoid confusion and delay in an anaphylaxis emergency… It is what children with food allergy and their families deserve’.^31^ Five years later, how many more children need to die in UK schools before this is implemented in the UK?^41^

## Supplementary material

10.1136/archdischild-2025-329493 online supplemental file 1

## Data Availability

Data may be obtained from a third party and are not publicly available.
