# Supplementary material for: Economic modelling of providing ‘spare’ adrenaline autoinjectors to all schools to improve the management of anaphylaxis
Source: Arch Dis Child. 2025 Oct 21;111(3):e329493. doi: 10.1136/archdischild-2025-329493 (PMC13018742; doi:10.1136/archdischild-2025-329493)
Supplement: online supplemental file 1 [file archdischild-111-3-s001.pdf]

Title page

Economic modelling of providing "spare" adrenaline autoinjectors to all schools to improve the management of anaphylaxis

Authors:

Paul J Turner,[1] Andrew D Bright,[2,3] Louise J Michaelis,[3,4] Jennifer K. Quint[5]

ONLINE SUPPLEMENT

Supplementary Methods

The analyses of the estimated number of school age children receiving AAIs and associated quantities and costs are based on electronic and paper-based NHS prescriptions dispensed in the community in England. This does not include medicines used in secondary care, prisons, issued by a private prescriber, or over-the-counter. The dataset was used to estimate the number of school-aged children in school years Reception to Year 11, who received AAI prescriptions (coded as per Table S1) during the academic years 2023/24 and 2024/25 (incomplete, data only available to March 2025). The dataset also quantifies the cost of the prescriptions, as well as the estimated quantity and associated costs for patients receiving more than two AAIs. The dataset is aggregated at a national level, ICB level and by patient IMD.

BNF Presentation Code	BNF Presentation Description
0304030C0AAA2A2	Adrenaline (base) 150mcg/0.3ml (1in2,000) inj pf dispos dev
0304030C0AAA3A3	Adrenaline (base) 300mcg/0.3ml (1in1,000) inj pf dispos dev
0304030C0AABFBF	Adrenaline (base) 150mcg/0.15ml (1in1,000) inj pf dispos dev
0304030C0AABGBG	Adrenaline (base) 500mcg/0.5ml (1in1,000) inj pf dispos dev
0304030C0BEAAA2	EpiPen Jr. 150microg/0.3ml (1 in 2,000) inj auto-injectors
0304030C0BEABA3	EpiPen 300micrograms/0.3ml (1 in 1,000) inj auto-injectors
0304030C0BHAABF	Jext 150micrograms/0.15ml (1 in 1,000) inj auto-injectors
0304030C0BHABA3	Jext 300micrograms/0.3ml (1 in 1,000) inj auto-injectors
0304030C0BIAABF	Emerade 150micrograms/0.15ml (1 in 1,000) inj auto-injectors
0304030C0BIABA3	Emerade 300micrograms/0.3ml (1 in 1,000) inj auto-injectors
0304030C0BIACBG	Emerade 500micrograms/0.5ml (1 in 1,000) inj auto-injectors

Table S1: BNF Prescription codes used in this analysis.

Data caveats

The following caveats apply to all NHSBSA data:

- NHSBSA data cannot confirm why a patient received a prescription nor what the remaining shelf life of their medication was when they received their prescription. NHSBSA prescriptions data can show the number of school aged children who have received an AAI dispensed in the community, the number of AAIs received and the cost of these AAIs. NHSBSA data does not show why the AAI were prescribed to a patient, for example, whether they are replacing expired pens, used pens, misplaced pens or whether they are

receiving additional pens to be kept in school. It also does not show instances where pens were dispensed in the hospital.

- Data are limited to prescriptions where an NHS number has been captured during NHSBSA processing. The proportion of AAI prescription items across the whole population that could not be linked to individual patients was 1.3% between August 2023 and March 2025. This means that data used in the analysis represents most, but not all, patients.
- Data are limited to prescribing in primary care in England which is dispensed in the community in England.
- Prescriptions excluded from the data are:
  - Items not dispensed, disallowed and those returned to the contractor for further clarification.
  - Prescriptions issued and dispensed in Prisons, Hospitals and through private practice.
  - Items prescribed but not presented for dispensing or not submitted to NHS Prescription Services by the dispenser.

The following caveats apply to the data used in the analysis:

- When a child reaches the recommended switching age, their prescribed dose may increase from 150mcg to 300mcg/500mcg. In such cases, only the updated stronger doses are considered for analysis. Conversely, if a child receives a 300mcg dose followed by a 150mcg dose, we still count only the devices linked to the most recent dose. This approach ensures we do not count any devices dispensed prior to any changes in a patient's prescribing regime.
- Data are limited to AAI prescriptions which were submitted to the NHSBSA for payment between August 2023 and March 2025. Prescribing intervals are: 12 months (Aug 2023–Jul 2024) and 8 months (Aug 2024–Mar 2025). The month in NHSBSA data relates to the dispensing month for which the prescription batch was submitted. This is generally, but not always, the month in which the prescription was dispensed. This means that there may be dispensing that has not been submitted to the NHSBSA for payment and is therefore not included.
- For the Academic year 2024/25, the 8-month time interval captures children between Reception and Year 11. Patients were only included if their date of birth was between 1st

September 2008 and 31st August 2020. The 12-month time interval during the 2023/24 academic year captured children between Reception and Year 11 only. Patients are only included in these analyses if their date of birth was between 1st September 2007 and 31st August 2019.

  - As these are estimated quantities, figures are rounded up or down to the nearest 10 throughout to reflect a degree of uncertainty.
  - School type reporting is based on the school year a child would have been in during the 23/24 or 24/25 academic year (depending on the chosen time interval), with Reception – Year 6 grouped together as Primary and Year 7 to Year 11 grouped as Secondary.

- ICB reporting is based on the ICB of the practice from which a patient received most of their AAI devices. If a patient's practice is not captured during processing, they are omitted from the ICB reporting analysis.
- Deprivation reporting is based on the patient's residential address supplied from their latest auto adrenaline injector prescription. Where address data wasn't captured directly from the prescription data, we have assigned the address based on other available data for the patient from other prescriptions and Personal Demographic Service (PDS). If no residential address information is available for a patient, they are omitted from the deprivation analysis.

Analysis was limited to the following metrics:

- Number of individuals with AAI: The estimated number of patients based on the count of the unique pseudonymised patient IDs who were dispensed an AAI in the given time period.
- Total number of AAIs dispensed: The sum of AAI devices received by patients.
- Estimated Total Cost of AAIs plus Applicable Fees: The sum of the Net Ingredient Cost (NIC, this relates solely to the basic price of the devices, in the quantity prescribed on a prescription form) plus the applicable prescription charge fees. Applicable fees included are professional fees and consumable container allowance.
- Estimated Cost of Supplying More than 2 AAIs: Determined by summing the total NIC plus fees cost, for all less two AAIs prescribed to a patient. The amount calculated is based on

the patient’s average AAI cost, multiplied by the number of AAI devices they received less 2.

- Estimated Proportion of Patients with ≥3 AAIs: The estimated number of patients, based on the number of unique pseudonymised patient IDs, where the patient received 3 or more devices divided by the total number of unique pseudonymised patient IDs who received any quantity of AAIs, then multiplied by 100.
- Estimated Proportion of Patients with ≥4 AAIs: The estimated number of patients, based on the number of unique pseudonymised patient IDs, where the patient received 4 or more devices divided by the total number of unique pseudonymised patient IDs who received any quantity of AAIs, then multiplied by 100.

Table S1: Provision of AAI in children aged 5-18 years in CPRD Aurum

	Overall cohort	5-10 years	11-18 years
**Probable food allergy**	**21,586**	**10,721**	**10,865**
• At least 1 AAI prescribed	9567 (44%)	5204 (49%)	4363 (40%)
• Repeat prescription for AAI	7300 (34%)	4262 (40%)	3038 (28%)
**Nut allergy specified**	**14,277**	**6910**	**7367**
• At least 1 AAI prescribed	7441 (52%)	3993 (58%)	3448 (47%)
• Repeat prescription for AAI	5727 (40%)	3292 (48%)	2435 (33%)
**Previous anaphylaxis specified**	**996**	**326**	**670**
• At least 1 AAI prescribed	584 (59%)	220 (68%)	364 (54%)
• Repeat prescription for AAI	436 (44%)	191 (59%))	245 (37%)

Table S2: Sensitivity analysis evaluating the impact of using 8 months of data (August-March) to estimate potential annual savings for the 12 months between August-July in each academic year of interest.

School Type		Number of individuals with AAI	Total number of AAIs dispensed	Estimated total cost of AAIs + applicable fees	Individuals with ≥3 AAIs		Individuals with ≥4 AAIs		Estimated annual cost of supplying "spare" AAI to schools	Estimated potential annual savings			
										Minimum		Maximum	
					%	Estimated cost of supplying >2 AAIs	%	Estimated cost of supplying >2 AAIs		£ million	% total budget on AAIs	£ million	% total budget on AAIs
**Academic year 2023/24 (children in reception to year 11): †**													
All	8m	63,390	219,740	£ 12.2m	54%	£ 5.2m	50%	£ 5.1m	£ 4.5m				
	12m[§]	95,085[§]	329,610[§]	£ 18.2m[§]		£ 7.8m[§]		£ 7.6m[§]		£ 3.1m[§]	17%[§]	£ 3.3m[§]	18%[§]
	12m	81,930	327,020	£ 18.3m	63%	£ 9.2m	60%	£ 9.0m		£ 4.6m	25%	£ 4.7m	26%
Primary	8m	37,210	133,430	£ 7.4m	58%	£ 3.3m	55%	£ 3.2m	£ 3.8m				
	12m[§]	55,815[§]	200,145[§]	£ 11.1m[§]		£ 4.9m[§]		£ 4.8m[§]		£ 1.0m[§]	9%[§]	£1.1m[§]	10%[§]
	12m	47,330	197,020	£ 11.0m	68%	£ 5.8m	64%	£ 5.7m		£ 1.9m	17%	£ 2.0m	18%
Secondary	8m	26,170	86,310	£ 4.8m	49%	£ 1.9m	44%	£ 1.8m	£ 0.7m				
	12m[§]	39,255[§]	129,465[§]	£ 7.2m[§]		£ 2.9m[§]		£ 2.8m[§]		£ 2.1m[§]	29%[§]	£ 2.2m[§]	31%[§]
	12m	34,590	130,000	£ 7.3m	57%	£ 3.4m	53%	£ 3.3m		£ 2.6m	36%	£ 2.7m	37%
**Academic year 2024/25 (children in reception to year 11): ‡**													
All	8m	67,410	237,810	£ 14.8m	56%	£ 6.5m	53%	£ 6.3m	£ 5.0m				
	12m[§]	101,115[§]	356,715[§]	£ 22.2m[§]		£ 9.7m[§]		£ 9.5m[§]		£ 4.5m[§]	20%[§]	£ 4.8m[§]	21%[§]
Primary	8m	39,290	143,610	£ 9.0m	60%	£ 4.1m	57%	£ 4.0m	£ 4.2m				
	12m[§]	58,935[§]	215,415[§]	£ 13.4m[§]		£ 6.1m[§]		£ 6.0m[§]		£ 1.8m[§]	14%[§]	£ 2.0m[§]	15%[§]
Secondary	8m	28,120	94,200	£ 5.9m	51%	£ 2.4m	47%	£ 2.3m	£ 0.8m				
	12m[§]	42,180[§]	141300[§]	£ 8.8m[§]		£ 3.6m[§]		£ 3.5m[§]		£ 2.7m[§]	31%[§]	£ 2.8m[§]	32%[§]

[§]estimated for 12 months, using data for an 8 month period August–March in the academic year of interest.

†omits 2.7% of items and their associated drug cost where a change in prescribing regime has been observed.

‡omits 3.4% of items and their associated drug cost where a change in prescribing regime has been observed.

Table S3: Economic cost of providing "spare" adrenaline autoinjectors to all schools in England, if this cost was offset by the cost of no longer routinely dispensing more than 2 devices to school-aged children, for the Academic year 2023/24, by ICB.* †

ICB	Number of individuals with AAI	Total number of AAIs dispensed	Estimated total cost of AAIs + applicable fees	Individuals with ≥3 AAIs		Individuals with ≥4 AAIs		Number of schools in ICB	Estimated annual cost of supply for "spare" AAI to schools	Estimated potential annual savings			
										Minimum		Maximum	
				%	Estimated cost of supplying >2 AAIs	%	Estimated cost of supplying >2 AAIs			£	% total budget on AAIs	£	% total budget on AAIs
NHS Bath and NE Somerset, Swindon and Wiltshire	830	3,050	£ 170,1k	59%	£ 79,3k	54%	£ 77,1k	390	£ 87,3k	(£ 10,2k)	-6%	(£ 7,9k)	-5%
NHS Bedfordshire, Luton and Milton Keynes	1,720	6,120	£ 342,1k	57%	£ 151,7k	52%	£ 147,3k	340	£ 76,1k	£ 71,2k	21%	£ 75,7k	22%
NHS Birmingham and Solihull	2,970	11,200	£ 627,4k	56%	£ 297,4k	49%	£ 286,7k	449	£ 100,5k	£ 186,3k	30%	£ 196,9k	31%
NHS Black Country	1,760	6,840	£ 382,8k	59%	£189,0k	55%	£ 184,5k	406	£ 90,8k	£ 93,7k	24%	£ 98,1k	26%
NHS Bristol, N Somerset and S Gloucestershire	890	3,040	£ 170,8k	54%	£ 72,6k	48%	£ 69,8k	307	£ 68,7k	£ 1,1k	1%	£ 3,9k	2%
NHS Buckinghamshire, Oxfordshire and Berkshire West	3,630	14,390	£ 803,1k	65%	£ 400,9k	60%	£ 391,9k	684	£ 153,1k	£ 238,9k	30%	£ 247,8k	31%
NHS Cambridgeshire and Peterborough	1,220	4,490	£ 250,0k	59%	£ 116,9k	54%	£ 113,5k	323	£ 72,3k	£ 41,2k	16%	£ 44,6k	18%
NHS Cheshire and Merseyside	2,100	8,490	£ 475,7k	65%	£ 242,8k	57%	£ 233,8k	900	£ 201,4k	£ 32,4k	7%	£ 41,4k	9%
NHS Cornwall & Isles of Scilly	570	2,130	£ 118,9k	60%	£ 56,3k	58%	£ 55,7k	267	£ 59,7k	(£ 4,0k)	-3%	(£ 3,5k)	-3%
NHS Coventry and Warwickshire	1,360	5,250	£ 293,8k	63%	£ 142,4k	58%	£ 139,0k	337	£ 75,4k	£ 63,6k	22%	£ 67,0k	23%
NHS Derby and Derbyshire	930	3,650	£ 204,1k	65%	£ 100,3k	62%	£ 99,1k	487	£ 109,0k	(£ 9,8k)	-5%	(£ 8,7k)	-4%
NHS Devon	1,260	4,650	£ 260,2k	60%	£ 120,8k	56%	£ 118,6k	474	£ 106,1k	£ 12,5k	5%	£ 14,8k	6%
NHS Dorset	920	3,710	£ 206,7k	71%	£ 104,6k	68%	£ 103,5k	224	£ 50,1k	£ 53,4k	26%	(£ 54,5k)	26%
NHS Frimley	1,700	6,030	£ 337,8k	55%	£ 149,7k	51%	£ 145,8k	239	£ 53,5k	£ 92,3k	27%	£ 96,2k	28%
NHS Gloucestershire	500	1,770	£ 98,69k	56%	£ 43,5k	52%	£ 42,3k	285	£ 63,8k	(£ 21,4k)	-22%	(£ 20,3k)	-21%
NHS Greater Manchester	2,670	11,840	£ 663,4k	66%	£ 366,3k	63%	£ 361,3k	1,023	£ 228,9k	£ 132,4k	20%	£ 137,4k	21%
NHS Hampshire and I. of Wight	2,650	11,510	£ 642,5k	74%	£ 347,3k	72%	£ 344,5k	600	£ 134,3k	£ 210,2k	33%	£ 213,0k	33%
NHS Herefordshire and Worcs	690	2,760	£ 154,1k	67%	£ 78,1k	61%	£ 75,8k	299	£ 66,9k	£ 8,9k	6%	£ 11,2k	7%
NHS Hertfordshire & W. Essex	3,420	11,410	£ 639,7k	47%	£ 258,8k	45%	£ 254,3k	591	£ 132,2k	£ 122,0k	19%	£ 126,5k	20%
NHS Humber and N. Yorkshire	1,740	6,860	£ 383,0k	64%	£ 190,3	60%	£ 186,3k	719	£ 160,9k	£ 25,5k	7%	£ 29,4k	8%

NHS Kent and Medway	2,450	9,170	£ 512,7k	60%	£ 240,3k	56%	£ 235,3k	654	£ 146,3k	£ 89,0k	17%	£ 94,0k	18%
NHS Lancashire & S. Cumbria	1,210	4,740	£ 265,2k	60%	£ 131,2k	57%	£ 129,0k	773	£ 173,0k	(£ 44,0k)	-17%	(£ 41,8k)	-16%
NHS Leicester, Leics and Rutland	1,850	6,620	£ 370,2k	55%	£ 165,5k	48%	£ 157,6k	395	£ 88,4k	£ 69,2k	19%	£ 77,1k	21%
NHS Lincolnshire	660	2,670	£ 149,4k	64%	£ 76,1k	59%	£ 74,4k	333	£ 74,5k	(£ 0,1k)	0%	£ 1,6k	1%
NHS Mid and South Essex	2,210	7,690	£ 430,0k	52%	£ 185,0k	49%	£ 181,0k	381	£ 85,3k	£ 95,8k	22%	£ 99,7k	23%
NHS Norfolk and Waveney	1,060	4,410	£ 245,1k	69%	£ 128,9k	65%	£ 126,6k	440	£ 98,5k	£ 28,2k	11%	£ 30,4k	12%
NHS North Central London	3,620	15,220	£ 853,3k	65%	£ 448,8k	63%	£ 444,3k	1,208	£ 270,3k	£ 174,0k	20%	£ 178,5k	21%
NHS NE London	4,330	18,080	£1,012,9k	62%	£ 530,6k	59%	£ 524,5k	372	£ 83,2k	£ 441,2k	44%	£ 447,4k	44%
NHS NE and N Cumbria	3,280	14,270	£ 797,4k	69%	£ 433,6k	66%	£ 428,5k	503	£ 112,6k	£ 316,0k	40%	£ 321,0k	40%
NHS NW London	4,970	19,970	£1,118,1k	65%	£ 565,8k	61%	£ 552,4k	491	£ 109,9k	£ 442,5k	40%	£ 456,0k	41%
NHS Northamptonshire	1,040	4,210	£ 233,6k	67%	£ 119,0k	63%	£ 116,8k	297	£ 66,5k	£ 50,3k	22%	£ 52,5k	22%
NHS Nottingham and Notts	1,270	5,100	£ 285,8k	63%	£ 143,4k	60%	£ 141,1k	420	£ 94,0k	£ 47,1k	16%	£ 49,4k	17%
NHS Shropshire, Telford & Wrekin	380	1,580	£ 88,1k	66%	£ 45,8k	66%	£ 45,8k	213	£ 47,7k	(£ 1,9k)	-2%	(£ 1,9k)	-2%
NHS Somerset	410	1,420	£ 79,5k	49%	£ 33,8k	49%	£ 33,8k	246	£ 55,0k	(£ 21,3k)	-27%	(£ 21,3k)	-27%
NHS SE London	4,350	19,200	£1,074,5k	72%	£ 589,3k	70%	£ 584,3k	486	£ 108,7k	£ 475,5k	44%	£ 480,5k	45%
NHS SW London	3,790	15,080	£ 844,5k	65%	£ 421,8k	61%	£ 414,5k	390	£ 87,3k	£ 327,2k	39%	£ 334,5k	40%
NHS S Yorkshire	1,400	5,890	£ 330,6k	65%	£ 175,3k	64%	£ 174,1k	477	£ 106,7k	£ 67,4k	20%	£ 68,5k	21%
NHS Staffordshire and Stoke-on-Trent	930	3,570	£ 199,4k	57%	£ 96,8k	54%	£ 95,1k	444	£ 99,3k	(£ 4,2k)	-2%	(£ 2,6k)	-1%
NHS Suffolk and NE Essex	1,280	4,800	£ 267,3k	59%	£ 125,9k	55%	£ 122,6k	375	£ 83,9k	£ 38,7k	14%	£ 42,0k	16%
NHS Surrey Heartlands	2,580	10,810	£ 604,1k	73%	£ 316,2k	70%	£ 311,7k	305	£ 68,2k	£ 243,5k	40%	£ 247,9k	41%
NHS Sussex	2,200	9,140	£ 510,5k	69%	£ 267,2k	65%	£ 262,7k	497	£ 111,2k	£ 151,5k	30%	£ 156,0k	31%
NHS West Yorkshire	3,190	14,190	£ 793,7k	71%	£ 439,0k	67%	£ 432,9k	875	£ 195,8k	£ 237,1k	30%	£ 243,2k	31%

*omits 3.4% of items and their associated drug cost where a change in prescribing regime has been observed.

†omits <0.01% of patients whose practice could not be aligned to an ICS.

Table S4: Economic cost of providing “spare” adrenaline autoinjectors to all schools in England, if this cost was offset by the cost of no longer routinely dispensing more than 2 devices to school-aged children, from August – March during the Academic year 2024/25, by ICB.* †

ICB	Number of individuals with AAI	Total number of AAIs dispensed	Estimated total cost of AAIs + applicable fees	Individuals with ≥3 AAIs		Individuals with ≥4 AAIs		Number of schools in ICB	Estimated annual cost of supply for “spare” AAI to schools	Estimated potential annual savings			
										Minimum		Maximum	
				%	Estimated cost of supplying >2 AAIs	%	Estimated cost of supplying >2 AAIs			£	% total budget on AAIs	£	% total budget on AAIs
NHS Bath and NE Somerset, Swindon and Wiltshire	720	2,410	£ 140,0k	56%	£ 61,3k	49%	£ 58,2k	390	£ 97,3k	(£ 10,0k)	-7%	(£ 5,3k)	-4%
NHS Bedfordshire, Luton and Milton Keynes	1,420	4,520	£ 281,7k	50%	£ 107,3k	45%	£ 103,0k	340	£ 84,8k	£ 69,7k	25%	£ 76,2k	27%
NHS Birmingham and Solihull	2,580	8,460	£ 528,0k	48%	£ 209,2k	41%	£ 197,3k	449	£ 112,0k	£ 184,0k	35%	£ 201,8k	38%
NHS Black Country	1,550	5,470	£ 340,8k	53%	£ 149,0k	47%	£ 143,4k	406	£ 101,2k	£ 113,8k	33%	£ 122,2k	36%
NHS Bristol, N Somerset and S Gloucestershire	740	2,300	£ 143,2k	45%	£ 51,5k	41%	£ 49,6k	307	£ 76,6k	(£ 2,1k)	-1%	£ 0,7k	0%
NHS Buckinghamshire, Oxfordshire and Berkshire West	2,870	9,970	£ 621,5k	57%	£ 264,9k	52%	£ 256,2k	684	£ 170,6k	£ 213,7k	34%	£ 226,8k	36%
NHS Cambridgeshire and Peterborough	920	3,070	£ 190,8k	52%	£ 78,0k	48%	£ 75,5k	323	£ 80,5k	£ 32,6k	17%	£ 36,4k	19%
NHS Cheshire and Merseyside	1,700	5,940	£ 370,4k	58%	£ 60,7k	51%	£ 153,8k	900	£ 224,4k	£ 6,3k	2%	£ 16,6k	4%
NHS Cornwall & Isles of Scilly	440	1,510	£ 93,7k	55%	£ 39,2k	50%	£ 37,9k	267	£ 66,6k	(£ 9,7k)	-10%	(£ 7,8k)	-8%
NHS Coventry and Warwickshire	1,160	3,850	£ 240,0k	53%	£ 97,1k	49%	£ 94,0k	337	£ 84,0k	£ 57,0k	24%	£ 61,6k	26%
NHS Derby and Derbyshire	740	2,430	£ 151,0k	51%	£ 60,0k	49%	£ 58,7k	487	£ 121,4k	(£ 33,4k)	-22%	(£ 31,5k)	-21%
NHS Devon	1,090	3,750	£ 233,7k	57%	£ 99,8k	54%	£ 97,9k	474	£ 118,2k	£ 28,7k	12%	£ 31,5k	13%
NHS Dorset	740	2,780	£ 173,0k	68%	£ 81,5k	65%	£ 80,3k	224	£ 55,9k	£ 64,6k	37%	£ 66,4k	38%
NHS Frimley	1,350	4,240	£ 264,5k	45%	£ 98,2k	40%	£ 93,9k	239	£ 59,6k	£ 81,2k	31%	£ 87,8k	33%
NHS Gloucestershire	400	1,300	£ 80,6k	53%	£ 31,5k	48%	£ 30,3k	285	£ 71,1k	(£ 25,6k)	-32%	(£ 23,8k)	-29%
NHS Greater Manchester	2,140	8,040	£ 501,2k	58%	£ 237,2k	54%	£ 231,6k	1,023	£ 255,1k	£ 92,3k	18%	£100,750	20%
NHS Hampshire and I. of Wight	2,170	8,440	£ 525,2k	68%	£ 256,5k	66%	£ 254,0k	600	£ 149,6k	£ 231,4k	44%	£235,149	45%
NHS Herefordshire and Worcs	540	1,910	£ 118,7k	59%	£ 51,3k	54%	£ 49,4k	299	£ 74,6k	£ 0,4k	0%	£2,391	2%
NHS Hertfordshire & W. Essex	2,760	8,130	£ 507,4k	39%	£ 165,3k	36%	£ 159,7k	591	£ 147,4k	£ 92,1k	18%	£100,548	20%
NHS Humber and N. Yorkshire	1,410	4,920	£ 305,9k	56%	£ 131,2k	53%	£ 128,7k	719	£ 179,3k	£ 13,8k	5%	£17,510	6%

NHS Kent and Medway	2,010	6,830	£ 425,3k	54%	£ 177,3k	50%	£ 173,0k	654	£ 163,1k	£ 96,4k	23%	£ 102,9k	24%
NHS Lancashire & S. Cumbria	980	3,520	£ 219,4k	53%	£ 98,0k	51%	£ 96,7k	773	£ 192,8k	(£ 47,7k)	-22%	(£ 45,8k)	-21%
NHS Leicester, Leics and Rutland	1,430	4,490	£ 280,1k	47%	£ 104,3k	38%	£ 96,8k	395	£ 98,5k	£ 46,7k	17%	£ 57,9k	21%
NHS Lincolnshire	520	1,770	£ 110,1k	52%	£ 46,7k	48%	£ 45,4k	333	£ 83,0k	(£ 14,9k)	-14%	(£ 13,0k)	-12%
NHS Mid and South Essex	1,830	5,620	£ 350,2k	44%	£ 125,9k	40%	£ 121,6k	381	£ 95,0k	£ 87,3k	25%	£93,889	27%
NHS Norfolk and Waveney	780	2,720	£ 169,1k	56%	£ 73,3k	55%	£ 72,7k	440	£ 109,7k	£ 0,7k)	0%	£ 0,2k	0%
NHS North Central London	3,100	11,510	£ 718,2k	60%	£ 332,2k	58%	£ 327,2k	1,208	£ 301,2k	£ 189,6k	26%	£ 197,1k	27%
NHS NE London	3,880	14,860	£ 927,2k	60%	£ 445,5k	57%	£ 437,4k	372	£ 92,8k	£ 563,3k	61%	£ 575,4k	62%
NHS NE and N Cumbria	2,470	9,020	£ 562,5k	59%	£ 255,9k	56%	£ 251,5k	503	£ 125,4k	£ 251,8k	45%	£ 258,3k	46%
NHS NW London	4,150	14,600	£ 910,7k	58%	£ 398,7k	54%	£ 387,4k	491	£ 122,4k	£ 458,7k	50%	£ 475,5k	52%
NHS Northamptonshire	840	2,840	£ 176,7k	54%	£ 73,2k	49%	£ 70,7k	297	£ 74,1k	£ 32,1k	18%	£ 35,8k	20%
NHS Nottingham and Notts	1,050	3,770	£ 235,2k	56%	£ 104,7k	54%	£ 103,4k	420	£ 104,7k	£ 50,4k	21%	£ 52,3k	22%
NHS Shropshire, Telford & Wrekin	310	1,050	£ 65,2k	55%	£ 27,5k	55%	£ 27,5k	213	£ 53,1k	(£ 11,8k)	-18%	(£ 11,8k)	-18%
NHS Somerset	380	1,160	£ 71,9k	45%	£ 25,7k	39%	£ 24,5k	246	£ 61,3k	(£ 24,6k)	-34%	(£ 22,8k)	-32%
NHS SE London	3,670	14,450	£ 901,3k	68%	£ 445,3k	66%	£ 441,0k	486	£ 121,2k	£ 540,3k	60%	£ 546,8k	61%
NHS SW London	3,160	11,300	£ 705,4k	59%	£ 313,9k	56%	£ 308,3k	390	£ 97,3k	£ 365,2k	52%	£ 373,6k	53%
NHS S Yorkshire	1,180	4,410	£275,4k	58%	£ 128,5k	55%	£ 126,0k	477	£ 118,9k	£ 70,1k	25%	£ 73,8k	27%
NHS Staffordshire and Stoke-on-Trent	780	2,560	£ 159,1k	49%	£ 63,4k	45%	£ 61,6k	444	£ 110,7k	(£ 18,4k)	-12%	(£ 15k,6)	-10%
NHS Suffolk and NE Essex	1,010	3,280	£ 203,4k	50%	£ 80,4k	46%	£ 77,9k	375	£ 93,5k	£ 23,4k	12%	£ 27,2k	13%
NHS Surrey Heartlands	2,010	7,390	£460,2k	64%	£ 210,6k	61%	£ 207,5k	305	£ 76,1k	£ 235,1k	51%	£ 239,8k	52%
NHS Sussex	1,750	6,330	£ 393,9k	59%	£ 177,4k	56%	£ 173,6k	497	£ 123,9k	£ 136,5k	35%	£ 142,1k	36%
NHS West Yorkshire	2,740	10,930	£ 681,2k	66%	£ 342,2k	63%	£ 337,2k	875	£ 218,2k	£ 287,6k	42%	£ 295,1k	43%

* omits 2.7% of items and their associated drug cost where a change in prescribing regime has been observed.

†omits <0.01% of patients whose practice could not be aligned to an ICS.

Table S5: Association between relative saving/cost of supplying "spare AAIs" by local demography

Factor	Pearson r	Estimate	95% CI	[t]	P value
▪ % urbanisation	0.61 (p<0.0001)	0.001046	-0.00142,0.00351	0.864	0.39
▪ Mean IMD	0.051 (p=0.75)	0.005336	-0.00130,0.0120	1.64	0.11
▪ % white British	-0.66 (p<0.0001)	-0.1321	-0.923, 0.659	0.339	0.74
▪ % Non-white	0.65 (p<0.0001)	-0.2297	-1.23, 0.767	0.469	0.64
▪ % with any AAI	0.76 (p<0.0001)	64.67	42.1, 87.3	5.83	<0.0001
▪ % with ≥3 AAIs	0.48 (p=0.0011)	1.529	-0.603, 3.66	1.46	0.15
▪ % with ≥4 AAIs	0.45 (p=0.0026)	-0.3541	-2.24, 1.53	0.382	0.70
▪ No. schools per 100,000 children (age 5-19 years)	-0.64 (p<0.0001)	-0.00181	-0.00239,-0.00124	6.43	<0.0001
